# Prognostic value of circulating tumor DNA and copy-number alterations in patients receiving tandem [^225^Ac]Ac-/[^177^Lu]Lu-PSMA-617 therapy for metastatic castration-resistant prostate cancer: a prospective observational study

**DOI:** 10.1186/s12885-026-16791-9

**Published:** 2026-08-29

**Authors:** Mariam Amghar, Thomas Hielscher, Tobias Rausch, Hilal Özgür, Ulrike Bauder-Wüst, Hendrik Rathke, Frank Bruchertseifer, Alfred Morgenstern, Mareike Roscher, Vladimír Beneš, Clemens Kratochwil, Martina Benešová-Schäfer

**Affiliations:** 1 Research Group Translational Radiotheranostics, German Cancer Research Center (DKFZ), Heidelberg, Germany; 2 Division of Biostatistics, German Cancer Research Center (DKFZ), Heidelberg, Germany; 3 Genomics Core Facility, European Molecular Biology Laboratory (EMBL), Heidelberg, Germany; 4 Cantonal Hospital Grisons, Chur, Switzerland; 5 Department of Nuclear Medicine, University Hospital, Heidelberg, Germany; 6 European Commission, Joint Research Centre (JRC), Karlsruhe, Germany; 7 Service Unit Radiopharmaceuticals and Preclinical Studies, German Cancer Research Center (DKFZ), Heidelberg, Germany

**Keywords:** ctDNA, PSMA-617, Radioligand therapy, Copy-number alterations, Tumor fraction

## Abstract

**Background:**

Prostate-specific membrane antigen-targeted radioligand therapy (PSMA-RLT) demonstrates clinical efficacy in metastatic castration-resistant prostate cancer (mCRPC), yet robust biomarkers for dynamic treatment monitoring and resistance remain lacking. We investigated circulating tumor DNA (ctDNA)-derived tumor fraction (TFx) and genome-wide copy-number alterations (CNAs) as non-invasive biomarkers of treatment response and resistance biology.

**Methods:**

Seventy-eight patients with advanced mCRPC receiving tandem [^225^Ac]Ac-/[^177^Lu]Lu-PSMA-617 were prospectively enrolled. Plasma samples collected longitudinally (*n* = 172) underwent ultra–low-pass whole-genome sequencing. TFx was estimated using ichorCNA, and recurrent CNAs were identified using GISTIC2.0. Associations with progression and overall survival (OS) were assessed using Cox proportional hazards models, including time-dependent analyses.

**Results:**

Baseline TFx differed across metastatic disease stages (*p* = 0.027) and dynamic TFx changes paralleled PSA kinetics during early treatment. Modelled as a time-dependent variable, TFx was associated with a significantly increased risk of progression (HR 4.9, 95% CI 1.2–20.1, *p* = 0.026). Unsupervised clustering identified distinct high- and low-CNA burden groups strongly correlated with TFx (*p* = 8.09 × 10⁻^8^). High CNA burden was associated with shorter median OS (8.3 vs 13.8 months). Multivariable analysis identified baseline logPSA and logALP as independent predictors of OS. Recurrent CNAs affected key tumor suppressors (PTEN, RB1, BRCA2, ATM) and were enriched in pathways related to TP53 signalling, homologous recombination repair, and oncogenic signaling. Longitudinal analyses demonstrated persistence and expansion of specific amplifications at progression.

**Conclusions:**

ctDNA-derived TFx represents a dynamic biomarker of treatment response and progression risk, while CNA profiling provides insight into resistance mechanisms in mCRPC treated with PSMA-RLT. These findings support the integration of ctDNA-based biomarkers into clinical stratification and real-time monitoring strategies.

**Supplementary Information:**

The online version contains supplementary material available at https://doi.org/10.1186/s12885-026-16791-9.

## Background

Management of metastatic castration-resistant prostate cancer (mCRPC) includes chemotherapy with docetaxel or cabazitaxel [1], androgen receptor-targeting agents such as abiraterone and enzalutamide [2, 3], and radioligand therapy (RLT) [4]. For patients harboring alterations in homologous recombination repair (HRR) genes, PARP inhibition such as olaparib an additional targeted treatment option [5]. Prostate-specific membrane antigen (PSMA)-targeted RLT with [^177^Lu]Lu-PSMA-617 (Pluvicto^®^) has transformed treatment for mCRPC patients and is currently FDA and Japan's PMDA-approved for those progressed on androgen receptor pathway inhibitors (ARPIs) and eligible to defer chemotherapy [6–8]. About 30% of patients develop resistance to [^177^Lu]Lu-PSMA-617 therapy, and although [^225^Ac]Ac-PSMA-617 has shown substantial activity, its use has been limited by irreversible toxicities [9, 10]. [^225^Ac]Ac-PSMA-617 has not reached the phase 3 trial stage, but several phase 2 trials are currently ongoing (NCT03276572, NCT04506567, NCT05219500, NCT04597411). Tandem therapy with [^177^Lu]Lu-PSMA-617 and [^225^Ac]Ac-PSMA-617 has shown promising results, even after progression on single-agent [^177^Lu]Lu-PSMA-617. Although early clinical results of tandem therapy are promising, patient stratification remains difficult and the molecular basis of resistance is largely unknown [11, 12]. Current monitoring relies on serum markers such as prostate-specific antigen (PSA), alkaline phosphatase (ALP), lactate dehydrogenase (LDH), which lack specificity and predictive value for PSMA-RLT [13, 14]. Circulating tumor DNA (ctDNA), the tumor-derived fraction of cell-free DNA (cfDNA), enables minimally invasive tracking of tumor burden, genomic alterations, and treatment response. Accumulating evidence highlights ctDNA fraction as a powerful prognostic biomarker in mCRPC, reflecting the genomic landscape of metastatic disease and correlating with tumor burden and clinical outcomes [15–17]. Tumor fraction (TFx), estimated using ichorCNA from ultra-low-pass whole-genome sequencing (ULP-WGS), provides a quantitative and dynamic measure of ctDNA levels and has emerged as a powerful prognostic biomarker in mCRPC [18]. Beyond quantifying tumor burden, ctDNA-based profiling enables detection of specific genomic aberrations including point mutations, insertions, deletions, and copy number alterations (CNAs)—changes that may directly drive treatment resistance [18–21]. Recent studies have demonstrated that copy number burden is associated with increased tumor aggressiveness and adversity in treatment response across multiple cancer types [22–25]. However, most prior studies in mCRPC have relied on tissue-based sequencing or single timepoint plasma analyses [26–28], limiting insight into longitudinal genomic evolution and mechanisms of acquired resistance. To address these limitations, we conducted a prospective longitudinal study integrating cfDNA-based genomic profiling with clinical outcomes in mCRPC patients undergoing PSMA-targeted radioligand therapy. While ctDNA-derived TFx is an established biomarker of treatment dynamics, its clinical utility in PSMA-targeted radioligand therapy and its relationship to resistance mechanisms remain poorly defined. In this study, we applied longitudinal TFx assessment alongside genome-wide CNAs profiling to evaluate their clinical relevance for patient stratification and to explore genomic mechanisms associated with treatment resistance in this distinct therapeutic setting.

## Methods

### Study design and participants

**Fig. 1:**
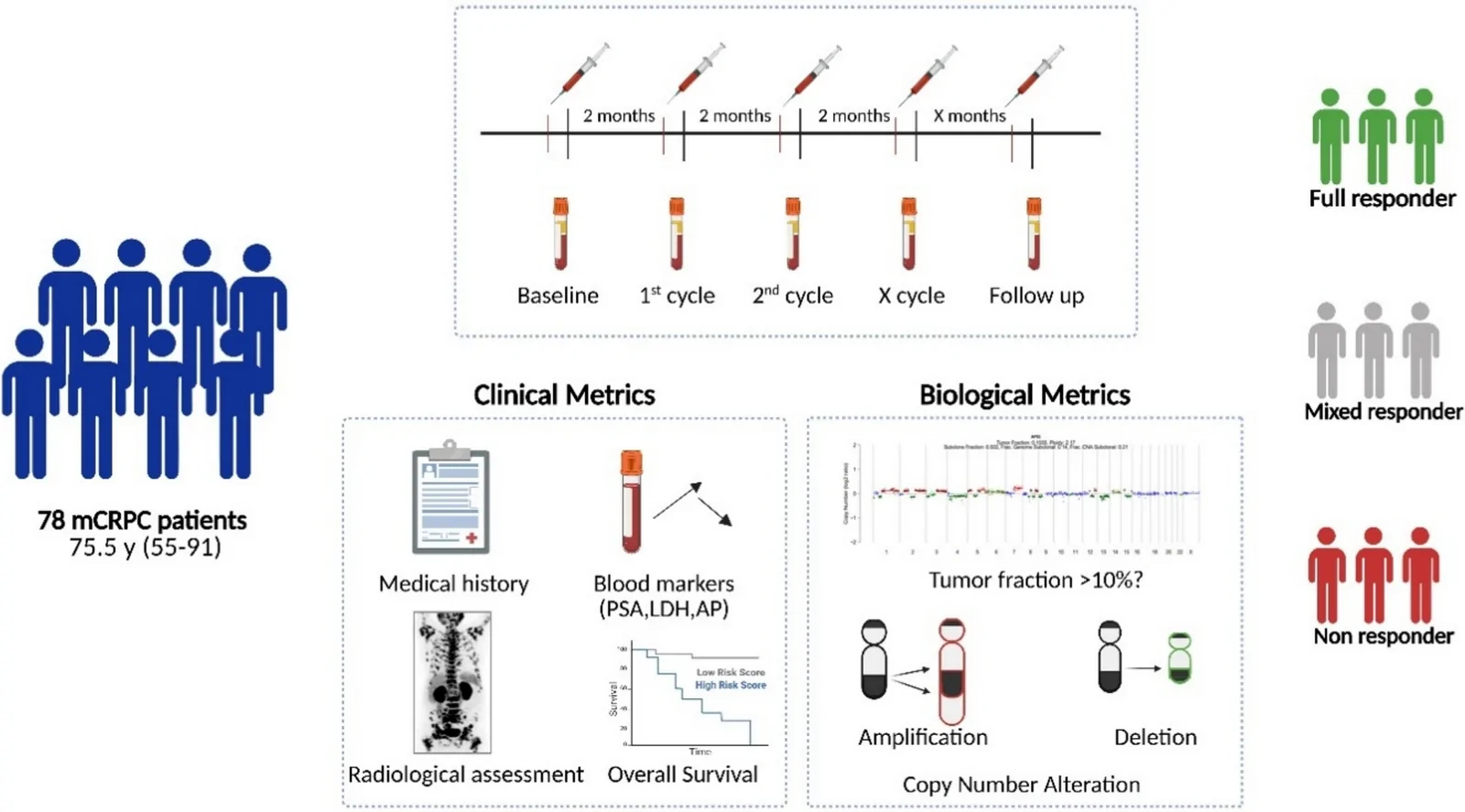
Overview of the study’s objective to stratify mCRPC patients undergoing PSMA-RLT. Overview of the bimonthly blood collection schedule prior to therapy administration, including the associated sample processing workflow. Clinical data, imaging, and plasma-derived cfDNA are analyzed to evaluate tumor burden and resistance. Patients are classified as responders, non-responders, or mixed responders, aiming to enhance treatment prediction and optimize therapeutic strategies. Schematic depiction of the molecular workflow from cfDNA extraction to sequencing

Eligible patients showed a PSMA-positive tumor phenotype as confirmed by imaging with either [^68^Ga]Ga-PSMA-11 and [^18^F]PSMA-1007 in PET(/CT), or [^99m^Tc]Tc-PSMA-GCK01 in SPECT. The selection of patients for [^225^Ac]Ac-/[^177^Lu]Lu-PSMA-617 therapy was primarily based on the presence of extensive bone marrow infiltration and diffuse liver or other organs metastases, which precluded the use of [^177^Lu]Lu-PSMA-617 (Pluvicto^®^) as stand-alone therapy [29]. The cohort included individuals with mCRPC who had exhausted all approved standard therapies, including second-generation androgen receptor inhibitors, taxane-based chemotherapy, or other unproven clinical interventions (Fig. 1). All patients provided written informed consent under ethics approval S-882/2020. [^225^Ac]Ac-/[^177^Lu]Lu-PSMA-617 was administered at approximately two-month intervals. Administered activities ranged from 1.5 to 7.4 GBq for [^177^Lu]Lu-PSMA-617 and from 4 to 6 MBq for [^225^Ac]Ac-PSMA-617. The rationale for the treatment regimen has already been described previously [12]. Details of imaging, inclusion criteria, and treatment regimens are provided in Supplementary data (Sect. "Background").

### Sample collection & processing

Venous blood was collected before each therapy cycle, and cfDNA was processed for ULP-WGS to derive genome-wide copy-number alterations, enabling longitudinal tracking of tumor burden across treatment. Venous blood samples (10–30 mL) were prospectively collected. Plasma was isolated, separated from the buffy coat, and stored at –80 °C. cfDNA was extracted using the QIAamp MinElute ccfDNA Midi Kit (Qiagen, Hagen, Germany; Cat. No./ID: 55284) following the manufacturer’s protocol. cfDNA library preparation was conducted using the Collibri PS DNA Library Prep Kit (Thermo Fisher, Karlsruhe, Germany). Libraries were pooled equimolarly, and paired -end (PE) sequenced (2 × 100 bp) on the Illumina NextSeq 2000 platform (Illumina Inc., San Diego, CA, USA). Data were analyzed with the ichorCNA algorithm (https://github.com/broadinstitute/ichorCNA) in R (v3.3.1) [18]. Wet-lab and bioinformatic pipelines are detailed in Supplemental data (Sects. "Methods"–"Results").

### Statistical analysis

Longitudinal trends in serum biomarkers (PSA, ALP, LDH) and TFx across treatment cycles were evaluated using Wilcoxon tests. Correlations between TFx and PSA, ALP, and LDH were assessed using Pearson correlation and linear mixed-effects models with patient-specific random effects [30]. Baseline PSA and TFx distributions were compared across metastatic disease categories (bone-only; bone + lymph nodes; bone + visceral involvement). Group differences were tested using Kruskal–Wallis analysis. Receiver operating characteristic (ROC) analyses evaluated the ability of baseline PSA, TFx, LDH, and a multivariable logistic regression model to predict clinical response at the subsequent treatment cycle. Clustered ROC analysis accounted for repeated measures per patient (Obuchowski (1997) [31]). Time-to-progression analyses were conducted using Cox proportional hazards models, treating TFx increases as time-dependent covariates. Genome-wide CNAs were quantified from logR values in 1 Mb bins derived from ichorCNA segment files. Baseline samples were hierarchically clustered (Ward’s D2 linkage) to stratify patients into low- and high-CNA burden groups. Group CNAs differences in TFx were assessed using Wilcoxon rank-sum testing. OS was analysed using Kaplan–Meier curves and Cox proportional hazards modelling. OS was defined as time from treatment initiation to death from any cause, with administrative censoring on November 7, 2025. Univariable and multivariable hazard ratios were computed using the R survival package. Recurrent CNAs were identified using GISTIC 2.0 [32], with broad-event modelling and predefined amplification/deletion thresholds. Genes within significant peaks were classified as oncogenes or tumor suppressors using COSMIC annotations. Functional enrichment was performed using EnrichR across Gene Ontology, KEGG, and Reactome databases.

## Results

### Cohort characterization

The study cohort comprised 78 mCRPC patients (median age 74.2 [53.9, 90.1]), from whom 172 longitudinal plasma samples were collected (Table 1). All patients subsequently received tandem [^177^Lu]Lu-/[^225^Ac]Ac-PSMA-617 RLT as study therapy. Patients were stratified according to their prior treatment history into three groups: chemotherapy + androgen receptor-targeting therapy (ART), chemotherapy + ART + PSMA-RLT, and chemonaïve. Among the 78 patients, 51.3% (40 patients) received chemotherapy + ART, 14.1% (11 patients) were chemonaïve and 34.6% (27 patients) received chemotherapy + ART + PSMA-RLT (Fig. S1). A deeper characterization of used radionuclides is presented in Figure S2. The largest segment, representing 48.7%, corresponds to patients who are RLT-naïve. 33.3% of the patients were treated with [^177^Lu]Lu-PSMA-617, making it the most frequently used radionuclide. 6.4% received [^223^Ra]RaCl_2_. A total of 5.1% of patients received combination radionuclide therapies as part of their pre-treatment. These included either a dual-treatment regimen with [^177^Lu]Lu-PSMA-617 and [^225^Ac]Ac-PSMA-617, or a combination of [^90^Y]Y-PSMA-617 with [^177^Lu]Lu-PSMA-617. The smallest segment, 1.3%, corresponds to patients treated with [^188^Re]Re-HYNIC-PSMA + [^177^Lu]Lu-PSMA-617 (Fig. S2).

**Table 1:** Patient baseline characteristics total cohort

**Characteristics**	**Overall (*n* = 78)**
Age	
Mean (SD)	73.4 (7.44)
Median [Min, Max]	74.2 [53.9, 90.1]
Pretreatment	
Chemotherapy + ART	39 (50.0%)
Chemotherapy + ART + RLT	28 (35.9%)
Chemotherapy-naïve	11 (14.1%)
Metastasis stage	
Bone	17 (21.8%)
Bone + Lymph nodes	45 (57.7%)
Bone + Lymph nodes + Additional organs	16 (20.5%)
BRCA mutational status	
Unknown	22 (28.2%)
Mutated	7 (9%)
Wild-type	49 (62%)
Overall response	
Mixed-responder	15 (19.2%)
Non-responder	20 (25.6%)
Responder	31 (39.7%)
Unknown	12 (15.4%)
Number of cycles	
Mean (SD)	2.21 (1.14)
Median [Min, Max]	2.00 [1.00, 7.00]
Number of cycles	
1	21 (26.9%)
2	34 (43.6%)
3	15 (19.2%)
4	4 (5.1%)
5	3 (3.8%)
6	2 (1.3%)

*ART* androgen receptor-targeting therapy, *RLT* radioligand therapy

### TFx dynamics mirror treatment response and correlate with clinical biomarkers

**Fig. 2:**
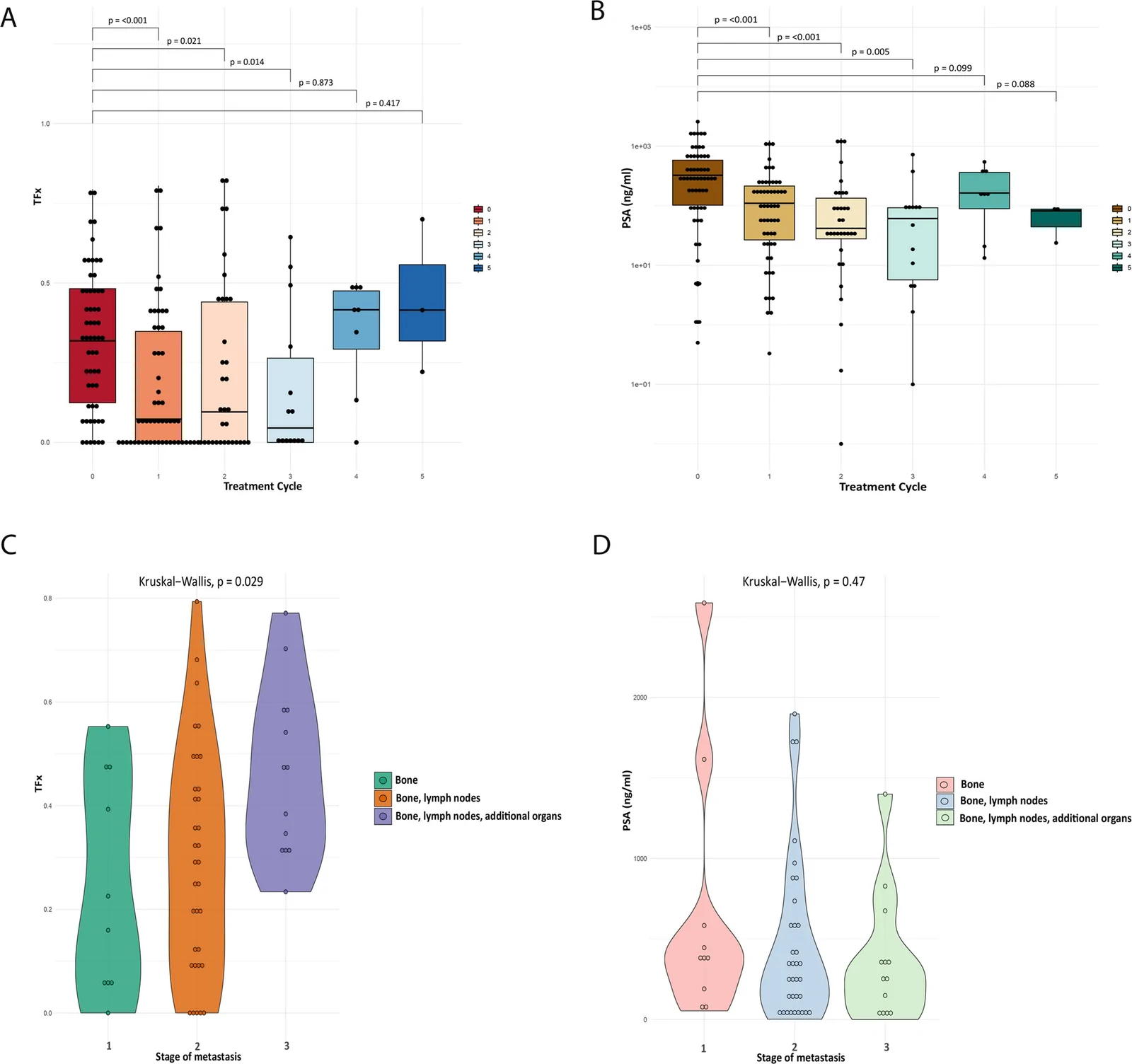
**A** TFx and **B** PSA (log₁₀ scale) across treatment cycles (cycle 0–5); box plots show median, IQR, and range; dots indicate individual patients. **C** Baseline TFx and D) PSA by metastasis site: bone-only, bone plus nodes, and bone plus nodes plus visceral involvement; violin width reflects density; dots indicate patients

TFx was evaluated at baseline (cycle 0) and across five subsequent [^225^Ac]Ac-/[^177^Lu]Lu-PSMA-617 treatment cycles (Fig. 2A). TFx dynamics closely reflected treatment response across therapy cycles, with significant reductions observed during early treatment (cycle 1: *p* < 0.001; cycle 2: *p* = 0.021; cycle 3: *p* = 0.014) followed by a rebound at later stages (cycle 4: *p* = 0.873; cycle 5: *p* = 0.417). These changes paralleled PSA kinetics (cycle 1: *p* < 0.001; cycle 2: *p* < 0.001; cycle 3: *p* = 0.005) (Fig. 2B), supporting TFx as a dynamic biomarker of treatment response. PSA levels plateaued thereafter, with no significant changes observed at later cycles (cycle 4: *p* = 0.099; cycle 5: *p* = 0.088). Significant reductions in ALP (*p* = 0.015) and LDH (*p* = 0.015), were observed only between baseline and cycle (Fig. S3, Fig. S4). The relationships between TFx and clinical biomarkers are summarized in Table S1. TFx levels showed a statistically significant positive correlation with PSA levels, as indicated by the upward trend of the regression line (*p* = 2.3 × 10⁻^10^) (Fig. S5), reflecting a moderate yet significant association. A highly significant association was observed between TFx and LDH levels (*p* = 6.3 × 10⁻^19^) (Fig. S6), indicating a robust relationship. Similarly, TFx correlated significantly with ALP levels (*p* = 4.2 × 10⁻^8^) (Fig. S7).

### TFx-PSA distribution across metastasis stages

Baseline TFx differed across metastatic sites involvement, with significant variation by stage (*p* = 0.029): patients with bone-only metastasis had the lowest TFx values, whereas those with visceral involvement showed the highest (Fig. 2C). In contrast, there is no clear distinction in PSA levels across metastatic sites (bone only; bone + lymph nodes; bone + lymph nodes + visceral; *p* = 0.47) (Fig. 2D).

### Time-to-event and predictive performance of TFx

**Fig. 3:**
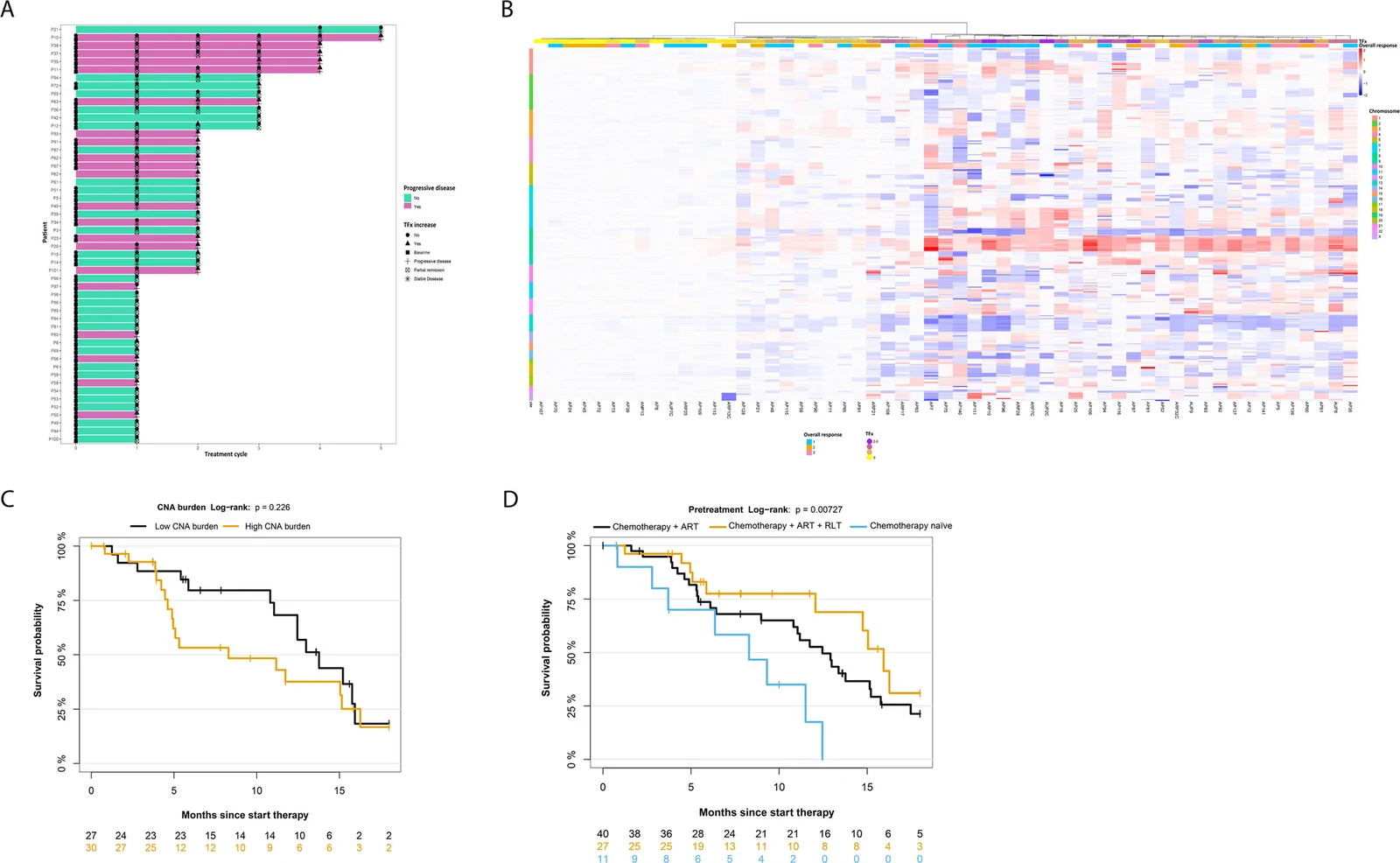
**A** Swimmer plot of individual treatment courses; bar color indicates outcome (green = non-progressive, pink = progressive); symbols mark TFx status and clinical annotations. **B** Heatmap of genome-wide CNAs (1 Mb bins; red = amplification, blue = deletion) with baseline TFx overlaid (purple = high, yellow = low) and treatment response (blue = responder, orange = mixed, pink = non-responder). **D** Kaplan–Meier curves stratified by CNA burden (low = black, high = yellow). Survival probability plotted over time (months since therapy initiation); numbers at risk indicated below. **B** Kaplan–Meier curves stratified by pre-treatment regimen: chemotherapy + ART (black), chemotherapy + ART + RLT (yellow), and chemotherapy-naïve (blue). Numbers at risk shown below

In time-to-event analysis, TFx demonstrated a strong prognostic value when modeled as a time-dependent variable. Increases in TFx were associated with a nearly five-fold higher risk of progression (HR 4.9, 95% CI 1.2–20.1, *p* = 0.026) (Fig. 3A). No meaningful discriminative potential was observed in the ROC analysis (Fig. S8).

### CNA clustering

Unsupervised clustering of CNA profiles from the 57 patients with available baseline samples (Table 2) revealed two distinct genomic subgroups characterized by low versus high CNA burden (Fig. 3B). CNA burden was strongly correlated with baseline TFx (*p* = 8.1 × 10⁻^8^) (Fig. S9).

**Table 2:** Patients characteristics based on the CNA clustering including only 57 patients with baseline sample available and sequenced

**Characteristics**	**Low CNA burden (*N* = 27)**	**High CNA burden (*N* = 30)**	**Overall (*N* = 57)**	***p*-value**
Age [years]				
Mean (SD)	74.1 (6.71)	71.3 (7.79)	72.7 (7.37)	0.228
Median [Min, Max]	74.2 [60.6, 90.1]	71.2 [53.9, 83.9]	73.8 [53.9, 90.1]	
Pretreatment				
Chemotherapy + ART	14 (51.9%)	15 (50.0%)	29 (50.9%)	1
Chemotherapy + ART + RLT	10 (37.0%)	12 (40.0%)	22 (38.6%)	
Chemotherapy naïve	3 (11.1%)	3 (10.0%)	6 (10.5%)	
Metastasis stage				
Bone	8 (29.6%)	2 (6.7%)	10 (17.5%)	0.023
Bone + lymph nodes	16 (59.3%)	18 (60.0%)	34 (59.6%)	
Bone + lymph nodes + additional organs	3 (11.1%)	10 (33.3%)	13 (22.8%)	
BRCA mutational status				
Unknown	10 (37.0%)	7 (23.3%)	17 (29.8%)	0.247
Mutated	1 (3.7%)	5 (16.7%)	6 (10.5%)	
Wild-type	16 (59.3%)	18 (60.0%)	34 (59.6%)	
Overall response				
Mixed-responder	8 (40.0%)	4 (16%)	12 (26%)	0.176
Non-responder	3 (15.0%)	8 (32%)	11 (24.4%)	
Responder	9 (45.0%)	13 (52%)	22 (48.9%)	
Unknown	7	5	12	
TFx				
Mean (SD)	0.164 (0.154)	0.469 (0.152)	0.325 (0.216)	< 0.001
Median [Min, Max]	0.121 [0, 0.541]	0.474 [0.209, 0.794]	0.319 [0, 0.794]	
PSA (ng/mL)				
Mean (SD)	353 (461)	589 (606)	477 (550)	0.058
Median [Min, Max]	190 [1.10, 1900]	345 [0.500, 2590]	326 [0.500, 2590]	
LDH (u/L)				
Mean (SD)	317 (175)	775 (855)	554 (663)	< 0.001
Median [Min, Max]	259 [195 997]	448 [192, 3680]	311 [192, 3680]	
ALP (u/L)				
Mean (SD)	330 (469)	453 (417)	394 (443)	0.012
Median [Min, Max]	138 [33.0, 1960]	289 [56.0, 1930]	232 [33.0, 1960]	

### Overall survival

Patients (Table 2) with low baseline CNA burden had a longer median OS than those with high CNA burden (13.8 vs 8.3 months; log-rank *p* = 0.112) (Fig. 3C). In multivariable analysis, CNA burden was not independently associated with overall survival (*n* = 56; HR 1.06, 95% CI 0.44–2.56, *p* = 0.89) (Table 3). Conversely, baseline logPSA and logALP emerged as independent prognostic factors, with consistent findings across both the CNA-assessed cohort (logPSA HR 1.38, 95% CI 1.04–1.83, *p* = 0.02; logALP HR 1.85, 95% CI 1.18–2.89, *p* = 0.0072) (Table 3) and full cohort analyses (*n* = 78; logPSA HR 1.31, 95% CI 1.05–1.64, *p* = 0.02; logALP HR 2.02, 95% CI 1.34–3.03, *p* = 0.00074) (Table 4). Stratification by pretreatment (Table 5) revealed significant differences in OS (*p* = 0.0073), with chemotherapy-naïve patients exhibiting the shortest survival (Fig. 3D). In both univariable (HR 2.74, 95% CI 1.19–6.32, *p* = 0.02) (Table 4) and multivariable analyses (HR 5.27, 95% CI 1.94–14.32, *p* = 0.0011) (Table 4), absence of prior chemotherapy was independently associated with increased risk of death. Patients with bone and lymph node metastases had worse outcomes (HR 2.69, 95% CI 1.12–6.48, *p* = 0.03), which further declined in those with additional visceral involvement (HR 5.26, 95% CI 1.74–15.86, *p* = 0.0032) (Table 4).

**Table 3:** Cox regression model CNA burden, multivariable

Multivariable model *N* = 56, events = 30	**coef**	**HR = exp (coef)**	**95% CI**	***p*-value**
CNA burden	0.06	1.06	[0.44, 2.56]	0.89
Log(PSA)	0.23	1.38	[1.04, 1.83]	0.02
Log(LDH)	0.11	1.17	[0.63, 2.17]	0.61
Log(ALP)	0.56	1.85	[1.18, 2.89]	0.0072
Age	−0.01	0.99	[0.93, 1.04]	0.64

**Table 4:** Cox regression model, univariable and multivariable

**Univariable model N = 78, events = 44**	**coef**	**HR = exp (coef)**	**95% CI**	***p*-value**
Chemotherapy + ART + RLT	−0.45	0.64	[0.31, 1.32]	0.23
Chemotherapy-naïve	1.10	2.74	[1.19, 6.32]	0.02

**Multivariable model N = 75, events = 42**	**coef**	**HR = exp (coef)**	**95% CI**	***p*-value**
Chemotherapy + ART + RLT	−0.65	0.52	[0.22, 1.26]	0.15
Chemotherapy-naïve	1.66	5.27	[1.94, 14.32]	0.0011
Log(PSA)	0.27	1.31	[1.05, 1,64]	0.02
Log(LDH)	−0.13	0.87	[0.50, 1.52]	0.63
Log(ALP)	0.70	2.02	[1.34, 3.03]	0.00074
Age	−0.03	0.97	[0.92, 1.01]	0.15
Bone + lymph nodes	0.99	2.69	[1.12, 6.48]	0.03
Bone + lymph nodes + additional organs	1.66	5.26	[1.74, 15.86]	0.0032

**Table 5:** Patients characteristics based on the pretreatment grouping including all 78 patients

Characteristics	Chemotherapy + ART (*N* = 40)	Chemotherapy + ART + RLT (*N* = 27)	Chemotherapy naïve (*N* = 11)	*p*-value
Age [years]				
Mean (SD)	71.1 (7.28)	73.9 (5.35)	80.6 (8.21)	0.10
Median [Min, Max]	71.5 [53.9, 84.5]	74.1 [63.3, 83.8]	81.1 [59.2, 90.1]	
Metastasis stage				
Bone	10 (25%)	5 (18.2%)	2 (18.2%)	0.84
Bone + Lymph nodes	22 (55%)	17 (63.6%)	6 (54.5%)	
Bone + Lymph nodes + Additional organs	8 (20%)	4 (18.5%)	3 (27.3%)	
BRCA mutational status				
Unknown	10 (25.0%)	9 (33.3%)	4 (36.4%)	0.46
Mutated	2 (5%)	3 (11.1%)	1 (9.1%)	
Wild-type	28 (70%)	15 (55.6%)	6 (54.5%)	
Total Prostatectomy				
No	23 (59%)	15 (55.6%)	10 (90.9%)	0.80
Yes	16 (41%)	10 (44.4%)	1 (9.1%)	
Unknown	1	0	0	
PSA (ng/mL)				
Mean (SD)	403 (511)	405 (471)	462 (636)	0.84
Median [Min, Max]	191 [1.99, 2590]	333 [0.500, 1900]	326 [1.13, 1760]	
LDH (u/L)				
Mean (SD)	572 (738)	454 (386)	403 (277)	0.84
Median [Min, Max]	309 [192, 3680]	299 [192, 2110]	327 [203, 1160]	
Missing	1		1	
ALP (u/L)				
Mean (SD)	419 (469)	278 (184)	346 (381)	0.94
Median [Min, Max]	210 [41, 1960]	234 [33, 757]	230 [85.0, 1350]	
Missing	2		1	

### GISTIC analysis of baseline ctDNA and validation in tissue cohorts

**Fig. 4:**
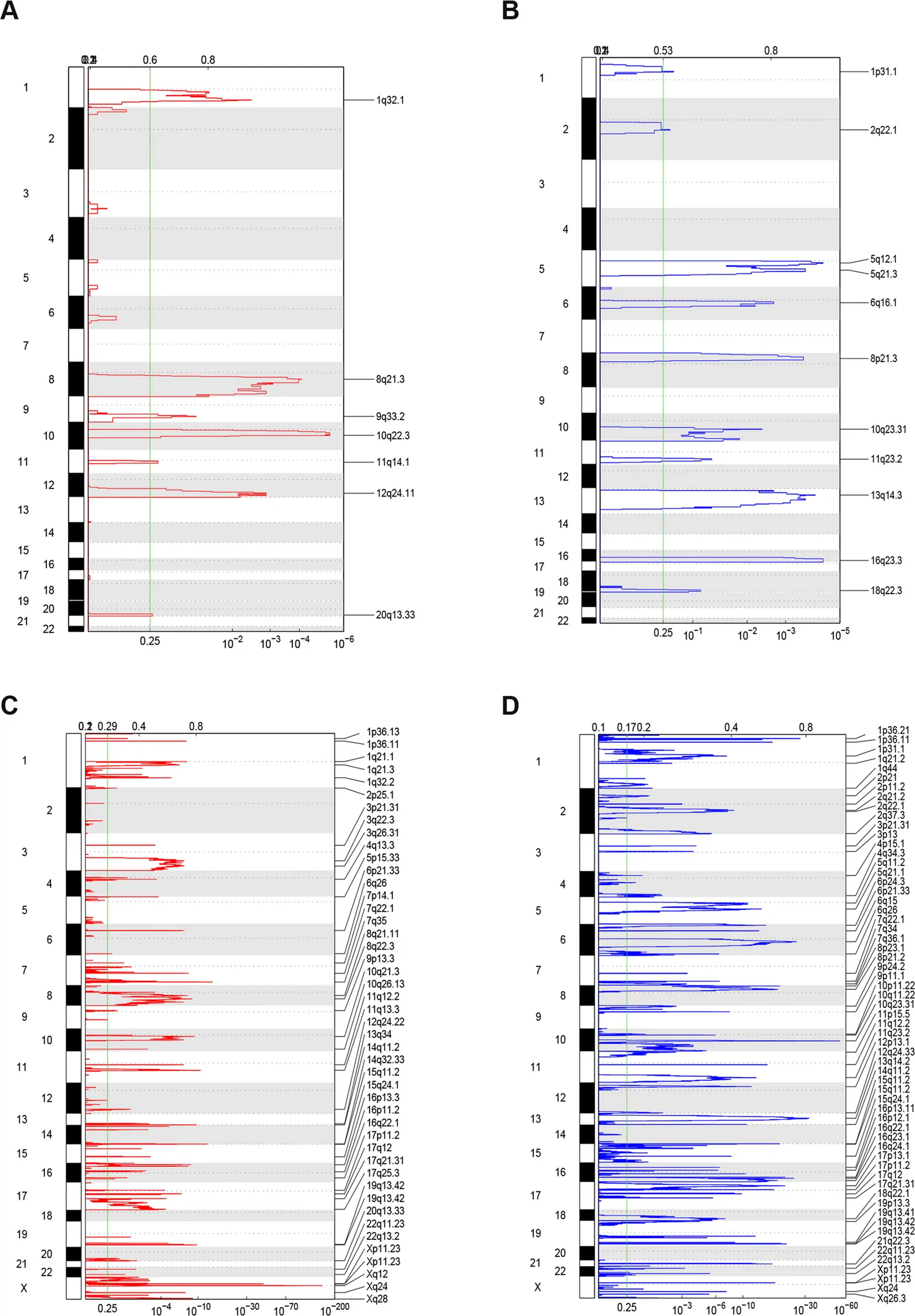
GISTIC analysis of genomic alterations in mCRPC. **A** Significant amplifications (red, right panel) and **B** deletions (blue, left panel) detected in cfDNA from 57 mCRPC patients. **C** Significant amplifications (red) and **D** deletions (blue) detected in tissue samples from the cBioPortal mCRPC dataset. Key loci are annotated in each panel. The top panels display G-scores, reflecting the magnitude of genomic alterations, while the bottom panels show q-values, indicating statistical significance. The green vertical line marks the significance threshold. Lower q-values correspond to higher statistical significance

GISTIC2.0 analysis of baseline ctDNA from 57 patients (Table 2) identified recurrent CNAs and deletions indicating genomic drivers of disease progression. Significant amplifications were found in chromosomal regions including 1q32.1, 8q21.3, 9q33.2, 10q22.3, 11q14.1, 12q24.11, and 20q13.33, whereas recurrent deletions occurred at 1q31.1, 2q22.1, 5q12.1, 5q21.3, 6q16.1, 8q21.3, 10q23.31, 11q23.2, 13q14.3, 16q23.3, and 18q22.3 (Fig. 4A–B). To validate the ctDNA-derived alterations, GISTIC analysis was performed on an independent cohort of 150 mCRPC tissue samples from the *SU2C/PCF* Dream Team dataset (2015) via cBioPortal (https://www.cbioportal.org/study/cnSegments?id=prad_su2c_2015) [33] (Fig. 4C–D).

### Genomic stratification by copy number burden identifies two distinct patient clusters

**Fig. 5:**
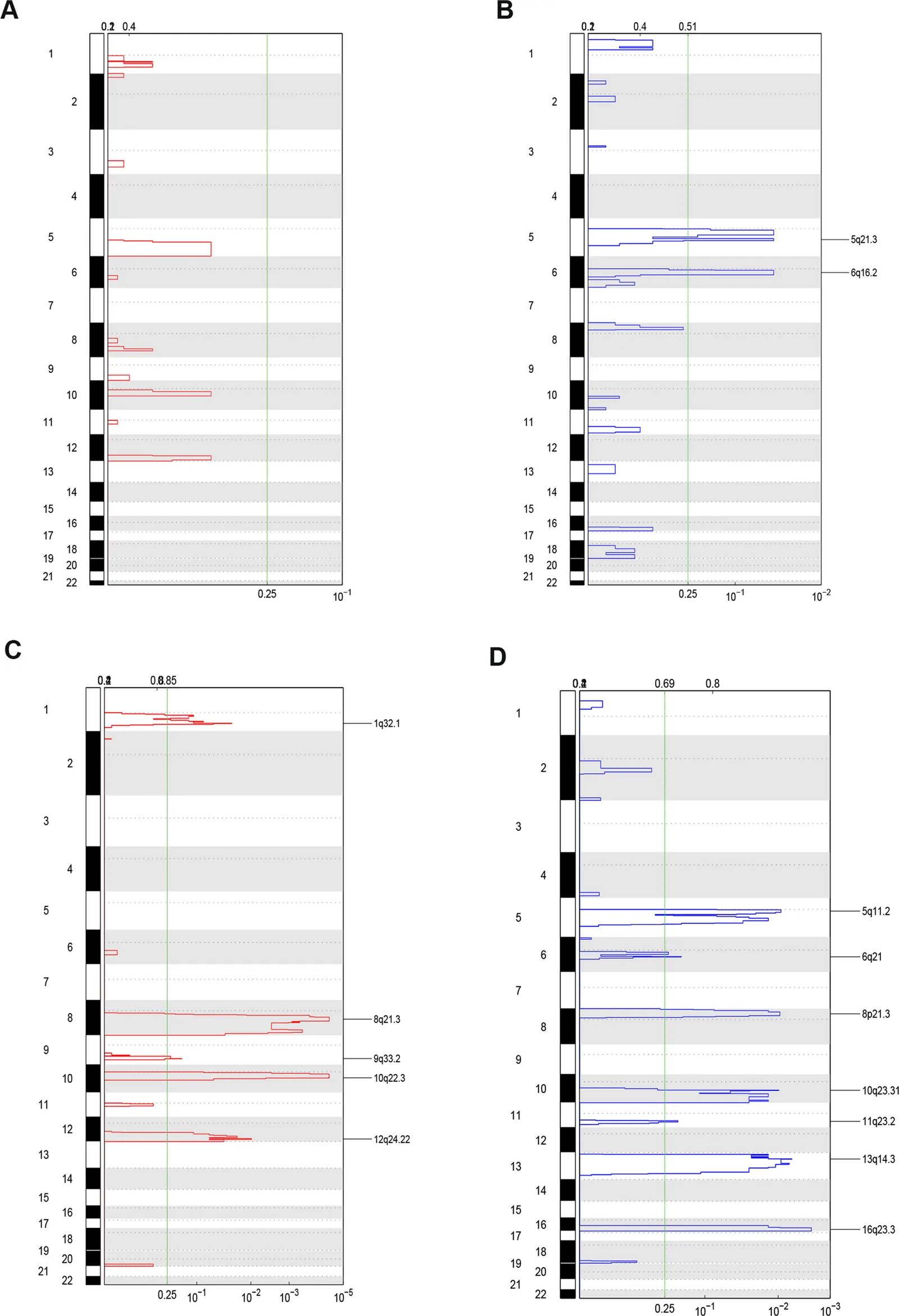
GISTIC analysis of amplifications and deletions in two mCRPC copy number alteration clusters. Panels **A**–**B** display Cluster 1 amplifications and deletions, while Panels **C**–**D** show Cluster 2 amplifications and deletions. Each panel contains two tracks: the upper track shows G-scores reflecting the magnitude of genomic alterations, and the lower track displays q-values indicating statistical significance. A vertical green line marks the significance threshold, with lower q-values denoting greater statistical significance. Key loci are annotated in each panel, highlighting recurrent driver alterations within each cluster

Cluster 1 (low CNA burden) demonstrated minimal genomic alterations, with no significant amplifications detected (Fig. 5A) and deletions restricted to 5q21.3 and 6q16.2 (Fig. 5B). In contrast, Cluster 2 (high CNA burden) exhibited markedly increased genomic complexity with frequent amplifications at 1q32.1, 8q21.3, 9q33.2, 10q22.3, and 12q24.22 (Fig. 5C), alongside deletions affecting 5q11.2, 6q21, 8p21.3, 10q23.31, 11q23.2, 13q14.3, and 16q23.3 (Fig. 5D).

### Longitudinal genomic changes from baseline to progressive disease

**Fig. 6:**
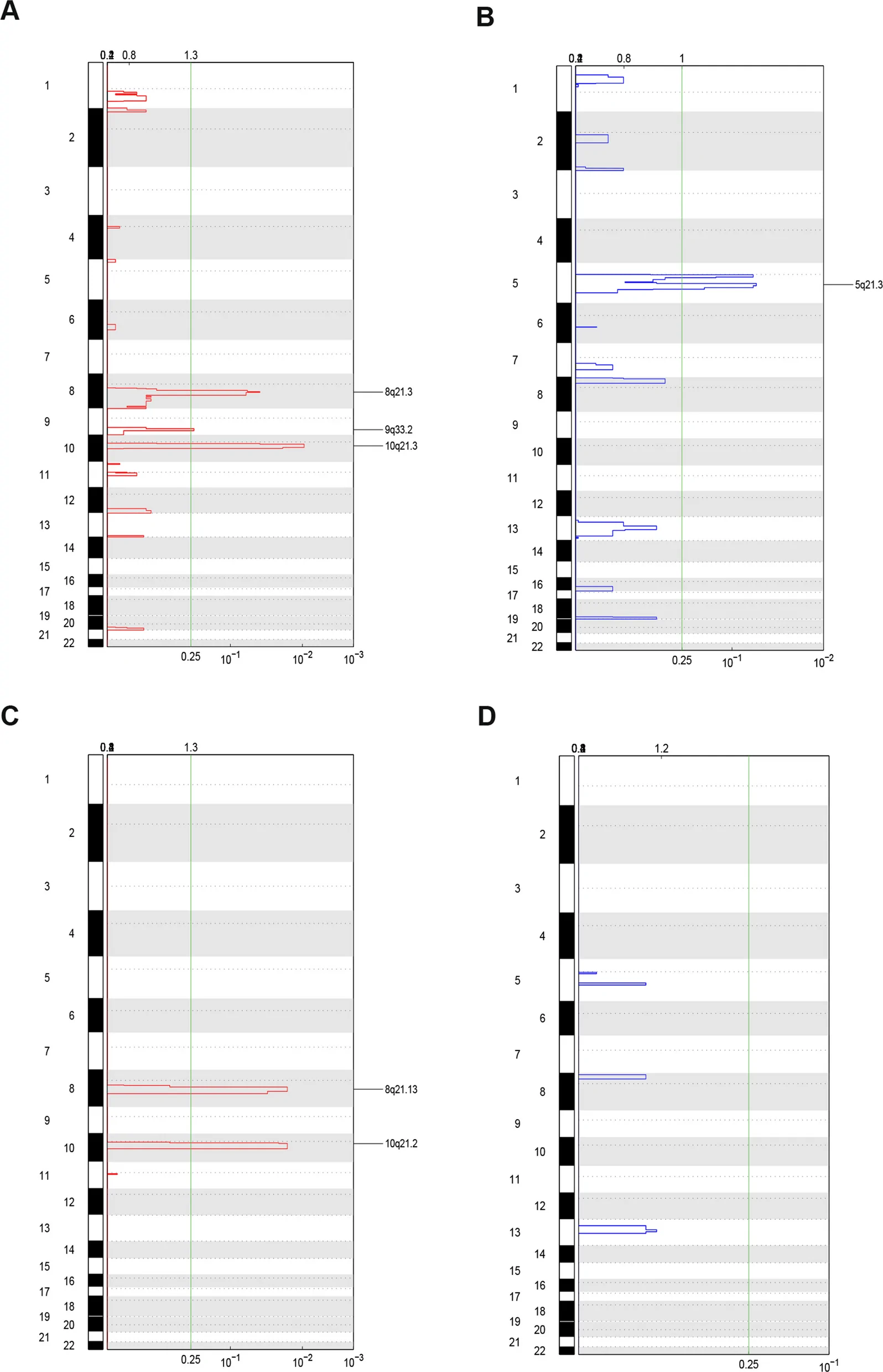
GISTIC analysis of amplifications and deletions in two mCRPC copy number alteration clusters. Panels **A**–**B** display 17 patients’ amplifications and deletions, while Panels **C**–**D** show progressive disease samples amplifications and deletions. Each panel contains two tracks: the upper track shows G-scores reflecting the magnitude of genomic alterations, and the lower track displays q-values indicating statistical significance. A vertical green line marks the significance threshold, with lower q-values denoting greater statistical significance. Key loci are annotated in each panel, highlighting recurrent driver alterations within each cluster

To identify CNA potentially associated with treatment resistance or clonal persistence, GISTIC analysis was performed on paired baseline and progressive disease (PD) samples from 17 mCRPC patients (filtering TFx ≥ 0.10). Baseline ctDNA demonstrated significant amplifications on 8q21.3, 9q33.2, and 10q21.3 (Fig. 6A), with a focal deletion on 5q21.3 (Fig. 6B). PD samples exhibited persistence of amplifications at 8q21.13 and 10q21.3 (Fig. 6C), with absent deletions (Fig. 6D) in the PD timepoint.

### Consensus gene-level alterations and pathway analysis

Gene-level analysis revealed consistent tumor suppressor deletions shared between baseline samples of the 17 patients who had matched PD samples and high CNA burden (Cluster 2) samples, supporting the robustness of the identified driver alterations. Common deletions included RB1, PTEN, PIK3R1, MGMT, FANCA, BRCA2, ATM, RAD17, PRDM1, LEPROTL1, FAS, SDHD, CCNC, CPEB3, and SUFU (Figure S10). In contrast, oncogene amplifications displayed a cluster-specific pattern. While CDH17 amplification was observed in both baseline and Cluster 2 samples, amplifications of MDM4, PTPN11, TNC and were only detected in Cluster 2. Pathway enrichment analysis of Cluster 2 revealed that oncogenes gains were associated with activation of tumor-promoting signaling, including cell–cell communication, MET–PTPN11, STAT5 activation, MAPK/ERK, NTRK, Netrin, IL-6, and leptin pathways (Fig. S11). Tumor suppressor losses were enriched for DNA damage repair and homologous recombination defects, including BRCA2–RAD51 interactions, TP53-mediated transcription, and homology-directed repair (Fig. S12).

## Discussion

This study systematically investigates the relationship between TFx, established clinical biomarkers, and genomic alterations in a well-characterized cohort of 78 mCRPC patients receiving [^225^Ac]Ac-/[^177^Lu]Lu-PSMA-617 as compassionate care. It aims to evaluate real-world clinical data, integrating patient stratification and TFx estimation as a biomarker to monitor treatment response and resistance. TFx kinetics closely mirrored and correlated to PSA-LDH-ALP, gold standards for response monitoring, supporting its potential as a surrogate biomarker for treatment response in mCRPC, as reported in the following case study [34]. The observed concordance between TFx dynamics and established clinical biomarkers (PSA, LDH, ALP) reinforces the biological validity of ctDNA-based monitoring, while also highlighting its advantages. In contrast to PSA, which may be unreliable in advanced disease [35, 36], TFx more accurately reflected metastatic burden and treatment dynamics. The strong association between TFx and LDH further supports its link to tumor metabolism and aggressive disease phenotypes [37, 38]. Consistent with this, PSA did not reliably distinguish metastatic burden, whereas TFx effectively did. In mCRPC patients treated with [^177^Lu]Lu-PSMA-617, baseline ctDNA levels have been linked to worse clinical outcomes [39]. Similarly, here, increased TFx was significantly associated with a higher risk of relapse. From a clinical perspective, these findings indicate that TFx may enable early identification of patients at risk of progression during PSMA-RLT, thereby supporting adaptive treatment strategies. Integration of ctDNA-based monitoring into clinical workflows could improve patient stratification and optimize therapeutic decision-making in this heterogeneous population. Although CNAs such as AR amplification are detectable across treatment courses, their prognostic value remains inconsistent across therapies, including abiraterone, enzalutamide, and [^177^Lu]Lu-PSMA-617 [40, 41]. Previous studies suggest that CNAs contribute most to the increase in the relative risk of disease progression and death [17, 19, 42]. While our real-world data have highlighted how ctDNA can be informative and impact RLT patients’ stratifications, this study is limited by the relatively small sample size and single-arm design. Although patients with high CNAs burden exhibited shorter median overall survival (8.3 vs 13.8 months), this difference was not statistically significant. However, the 5.5-month survival reduction represents a clinically meaningful difference that warrants further investigation. Sathekge et al. demonstrated a favorable safety profile of [^225^Ac]Ac-PSMA-617 in chemotherapy-naïve mCRPC patients [43]. Chemotherapy-naïve patients in our cohort had poorer OS. This group comprised few patients, highlighting the limited sample size. Patients with the longest survival had prior exposure to ART, chemotherapy, and RLT, regardless of the radionuclide used, consistent with literature [11]. Prior meta-analyses reporting shorter OS in [^177^Lu]Lu-PSMA-pretreated patients, our cohort shows longer OS in these patients [44]. ALP was a significant prognostic factor, confirming extensive evidence of baseline ALP as a robust mCRPC prognostic marker [45]. Although Ahmadzadehfar et al. demonstrated that baseline PSA was not independently prognostic in [^1^⁷⁷Lu]Lu-PSMA-617, our data reveal baseline logPSA as an independent predictor [46]. Patients with bone and lymph node metastases had worse outcomes, which further declined with visceral involvement consistent with recent data showing visceral metastases as the strongest independent predictor of poor OS in PSMA radioligand therapy [47]. Cluster 1 patients, characterized by a low CNA burden and as previously reported showed longest OS, exhibited chr 5q21.3 and chr 6q16.2 deletions with no significant amplifications. Cluster 2, characterized by a high CNA burden, showed genomic alterations associated with poorer OS, including amplification of chr 1q32.1. Similarly, chr 8q21.3 amplification, a frequently altered region in mCRPC, includes MYC, a key regulator of cell cycle progression and immune evasion, whose overexpression has been linked to increased tumor aggressiveness and therapy resistance [48, 49]. To identify specific alterations driving resistance, further analysis was conducted on 17 patients with PD, examining CNAs that persisted from baseline to PD timepoint. Amplifications at chr 8q21.3 and chr 10q21.2, enriched at baseline, progression, and in the high–CNA cluster, implicate these loci as drivers of tumor progression and resistance to [^225^Ac]Ac-/[^177^Lu]Lu-PSMA-617. Chromosome 8 amplification was also extensively observed in chemotherapy-naïve patients highlighting its potential role in disease progression [50]. The PROfound study demonstrated that ctDNA testing effectively identifies BRCA1, BRCA2, and ATM alterations, enabling targeted therapy decisions with PARP inhibitors for patients with defective DNA repair pathways [51]. BRCA2 deletions in Cluster 2 highlight defective homologous recombination repair (HRR), driving genomic instability, therapeutic resistance, and increased mutation burden. These finds are quite in contrast to what has been described in the literature. van der Doelen et al. observed pathogenic BRCA1 mutations in two patients associated with longer survival, suggesting that DDR-deficient tumors may exhibit higher PSMA expression and increased sensitivity to PSMA-RLT [52]. In contrast, our findings link DDR alterations to a more aggressive disease phenotype. Although DDR-deficient tumors may be more sensitive to RLT due to impaired repair of alpha-induced double-strand breaks, germline or somatic DDR alterations are also associated with higher PSMA expression, supporting their potential as biomarkers for PSMA-RLT [53, 54]. On the same setting, Slootbeek et al. described the impact of TP53 loss of function as a prognostic factor in PSMA-RLT outcomes in patients with mCRPC using tumor biopsies [28]. Our pathway enrichment analysis identifies dysregulation of TP53-mediated transcriptional programs in high-CNV burden tumors, providing direct molecular validation of Slootbeek et al.’s clinical observation that TP53 loss-of-function is the primary driver of poor PSMA-RLT response. Unlike tumor tissue, cfDNA reflects a composite genetic profile from multiple clonal populations, rather than a single tumor site.

## Conclusions

In conclusion, TFx effectively reflects treatment dynamics in mCRPC patients undergoing [^225^Ac]Ac-/[^177^Lu]Lu-PSMA-617 therapy and is associated with progression risk. In this real-world cohort, despite inherent clinical heterogeneity and potential confounding factors related to prior treatments and disease characteristics, TFx suggested consistent associations with clinical outcomes. These findings provide insight into optimizing tandem therapy administration, and prospective validation in larger, controlled cohorts is warranted to confirm clinical utility.

## Supplementary Information


Supplementary Material 1.


## Data Availability

The data generated in this study are available via controlled access in the German Human Genome-Phenome Archive (GHGA, data.ghga.de) under the GHGA Accession https://data.ghga.de/study/GHGAS41149685821148.
