# Supplementary material for: Prognostic value of circulating tumor DNA and copy-number alterations in patients receiving tandem [225Ac]Ac-/[177Lu]Lu-PSMA-617 therapy for metastatic castration-resistant prostate cancer: a prospective observational study
Source: BMC Cancer. 2026 Aug 29;26:1038. doi: 10.1186/s12885-026-16791-9 (PMC13531912; doi:10.1186/s12885-026-16791-9)
Supplement: Supplementary file 1 [file 12885_2026_16791_MOESM1_ESM.docx]

**Prognostic value of circulating tumor DNA and copy-number alterations in patients receiving tandem [^225^Ac]Ac-/[^177^Lu]Lu-PSMA-617 therapy for metastatic castration-resistant prostate cancer: a prospective observational study**

**Authors:** Mariam Amghar^1^, Thomas Hielscher^2^, Tobias Rausch^3^, Hilal Ozgur^3^, Ulrike Bauder-Wüst^1^, Hendrik Rathke^4,5^, Frank Bruchertseifer^6^, Alfred Morgenstern^6^, Mareike Roscher^7^, Vladimír Beneš^3^, Clemens Kratochwil^5^, Martina Benešová-Schäfer^1^.

**Institutes:** Translational Radiotheranostics^1^, Division of Biostatistics^2^, Genomics Core Facility, European Molecular Biology Laboratory (EMBL)^3^, Cantonal Hospital Grisons, Chur, Switzerland^4^, Department of Nuclear Medicine, University Hospital, Heidelberg, Germany^5^, European Commission, Joint Research Centre (JRC), Karlsruhe, Germany^6^, Service Unit Radiopharmaceuticals und Preclinical Studies^7^, German Cancer Research Center (DKFZ), Heidelberg, Germany^1,2,7^

**E-Mail addresses:**

[mariam.amghar@dkfz-heidelberg.de](mailto:mariam.amghar@dkfz-heidelberg.de); [t.hielscher@dkfz-heidelberg.de](mailto:t.hielscher@dkfz-heidelberg.de); [rausch@embl.de](mailto:rausch@embl.de); [hilal.ozgur@embl.de](mailto:hilal.ozgur@embl.de); [u.bauder-wuest@dkfz-heidelberg.de](mailto:u.bauder-wuest@dkfz-heidelberg.de); [hendrik@rathke.eu](mailto:hendrik@rathke.eu); [frank.bruchertseifer@ec.europa.eu](mailto:frank.bruchertseifer@ec.europa.eu); [alfred.morgenstern@ec.europa.eu](mailto:alfred.morgenstern@ec.europa.eu); [mareike.roscher@dkfz-heidelberg.de](mailto:mareike.roscher@dkfz-heidelberg.de); [benes@embl.de](mailto:benes@embl.de); [clemens.kratochwil@med.uni-heidelberg.de](mailto:clemens.kratochwil@med.uni-heidelberg.de); [m.benesova@dkfz-heidelberg.de](mailto:m.benesova@dkfz-heidelberg.de).

***Correspondence to:**

Dr. Martina Benešová-Schäfer

German Cancer Research Center (DKFZ)

Im Neuenheimer Feld 280

69120 Heidelberg

Germany

**E-mail**: [m.benesova@dkfz-heidelberg.de](mailto:m.benesova@dkfz-heidelberg.de); **Phone:** +49-6221-42-5355

**Supplementary data**

# **1. Study design and participants**

## **1.1 Ethical approval**

The study, titled *“Molekulare Charakterisierung der Tumorevolution im Rahmen von Endoradiotherapien des metastasierten Prostatakarzinoms “(“Molecular Characterization of Tumor Evolution in the Context of Endoradiotherapies for Metastatic Prostate Cancer”)*, received ethical approval *(S-882/2020)* from the Ethics Committee of the Medical Faculty at Heidelberg University. This project is a non-interventional, exploratory investigation focusing on therapy resistance in patients treated with [^225^Ac]Ac-/[^177^Lu]Lu-PSMA-617. The study complies with national regulations, the *Declaration of Helsinki*, and *Good Clinical Practice (GCP)* guidelines. Furthermore, in accordance with *Paragraph 13(2b) of the German Pharmaceuticals Law*, the therapy was administered as a salvage treatment for patients with mCRPC who had progressive disease and were either resistant to or ineligible for approved therapeutic options. Written informed consent was obtained from all participants prior to inclusion, ensuring that patients could withdraw their consent at any time without repercussions. The study involves the collection of blood samples from mCRPC patients scheduled for bimonthly [^225^Ac]Ac-/[^177^Lu]Lu-PSMA-617 cycles, with no additional risks or burdens to participants since it was taken in addition to the standard clinical blood collection to estimate clinical parameters and the eligibility of patients to receive the therapy. Subsequent to the blood collection, samples were pseudonymized to protect patient confidentiality in accordance with the *EU General Data Protection Regulation (GDPR)*. The protocol was designed to ensure compliance with ethical and legal standards, and the study does not involve any commercial sponsorship or conflicts of interest.

## **1.2 Patients**

This study encompasses a cohort of 78 patients who received [^225^Ac]Ac-/[^177^Lu]Lu-PSMA-617 under the conditions of the updated declaration of Helsinki, paragraph 37 (Unproven Interventions in Clinical Practice). The cohort included individuals with mCRPC who had exhausted all approved standard therapies (second-generation androgen receptor inhibitors, taxane-based chemotherapy, or other unproven clinical interventions). Following a comprehensive evaluation by the tumor board and in light of the absence of alternative treatment options, PSMA-directed RLT was proposed as a last-resort intervention for these patients. From the total cohort, 41 patients had available paired samples at baseline and one subsequent sample, 57 patients had only baseline samples, and 17 patients had baseline and progressive disease (PD) samples (Table S1). These subsets were selected for subsequent molecular and clinical analyses.

**2. Sample collection & processing**

Venous blood samples (10–30 mL) were prospectively collected in EDTA-coated tubes (BD Biosciences) and processed within 1 hour of collection upon the patients' arrival at the clinic, prior to undergoing therapy. Once collected, the clinic notified the research team, and the samples were transported from the Nuclear Medicine Department (Nuklearmedizin) of the University Hospital (Universitätsklinikum Heidelberg) to the DKFZ for processing. The samples underwent density gradient centrifugation at 2000 x g for 10 minutes at 4°C without braking to separate the plasma and buffy coat employing Heraeus Christ Minifuge GL 4400 centrifuge (Heraeus Christ, Osterode am Harz, Germany). The plasma was carefully isolated, transferred to 15 mL Falcon tubes, and separated from the buffy coat. Immediately after processing, the plasma samples were frozen at –80°C to ensure preservation until further analysis.

### **2.1 cfDNA extraction quantification and quality control**

cfDNA was extracted from thawed plasma aliquots using the QIAamp MinElute ccfDNA Midi Kit (Qiagen, Hagen, Germany; Cat. No./ID: 55284) following the manufacturer’s protocol. Quantification of the extracted cfDNA was performed using the Qubit 1X dsDNA High Sensitivity (HS) Assay Kit (Thermo Fisher, Karlsruhe, Germany) on a Qubit 4 Fluorometer (Thermo Fisher, Karlsruhe, Germany; Q33238). For DNA fragment analysis, including quantification, sizing, and purity determination, the High Sensitivity D1000 ScreenTape assay (Agilent, Waldbronn, Germany) was employed using 4150 TapeStation System (Agilent, Walbronn, Germany).

### **2.2 cfDNA library preparation**

The cfDNA samples were transported to the European Molecular Biology Laboratory GeneCore Facility, where library preparation and next-generation sequencing were conducted. cfDNA library preparation was conducted using the Collibri PS DNA Library Prep Kit (Thermo Fisher, Karlsruhe, Germany) for Illumina sequencing platforms (Illumina Inc., San Diego, CA, USA). Input cfDNA amounts ranged from 3–20 ng, with a size selection of 100–200 bp, optimized for ultra-low-pass whole genome sequencing (ULP-WGS). Following library preparation, quality control (QC) was performed using the Bioanalyzer High Sensitivity DNA Assay (Agilent, Waldbronn, Germany) to verify the fragment size distribution, targeting a peak at 300 bp. cfDNA concentrations were measured using the Qubit 4 Fluorometer (Thermo Fisher, Karlsruhe, Germany) using the Qubit High Sensitivity Kit (Thermo Fisher, Karlsruhe, Germany). Polymerase chain reaction (PCR) amplification was tailored to the quality of the cfDNA: optimal-quality samples underwent 8 cycles, while suboptimal-quality samples required 12 cycles.

## **3. Next Generation Sequencing**

Libraries were pooled equimolarly, and paired -end (PE) sequenced (2×100 bp) on on the Illumina NextSeq 2000 platform (Illumina Inc., San Diego, CA, USA) with sequencing kits selected based on the number of samples processed per time point. For 16 samples, the P2 200 cycles kit (100 PE), and for larger sample pools of up to 32 samples, the P3 200 cycles kit (100 PE) was employed. The number of reads generated per sequencing run varied depending on the flow cell kit used. Specifically, the P2 kit yielded around 400 million paired-end reads, while the P3 kit generated up to 1 billion paired-end reads. Given the human genome size of approximately 3 billion base pairs, resulting in mean theoretical coverage of ~1.67× for 16 samples (P2), and ~0.83× for 32 samples (P3). To ensure comparability between datasets generated from different kits and depths, coverage normalization was performed using Samtools to downsample all samples to a uniform depth suitable for ultra-low-pass whole genome sequencing (ULP-WGS) analysis using ichorCNA.

### **3.1 ULP-WGS data analysis**

ULP-WGS of cfDNA was performed, achieving an average genome-wide coverage of approximately 2x. From this data, segmental copy number alterations and TFx were estimated using the ichorCNA (*2,3*). In brief, the genome was segmented into non-overlapping bins of 1 megabase (Mb), and aligned sequencing reads were quantified within each bin using the HMMcopy Suite. To avoid artefacts, centromeric regions were excluded based on chromosome gap coordinates from the UCSC Genome Browser for hg38 (GRCh38), along with 1 Mb upstream and downstream flanking regions. Normalization of read counts was carried out using the HMMcopy R package, correcting for GC content and mappability biases. Log2 copy number ratios were then calculated for each bin by comparing them to a reference panel of ULP-WGS data from 27 healthy donors. Since cfDNA represents a mixture of tumor- and non-tumor-derived fragments, copy number calling and TFx estimation were performed using a hidden Markov model (HMM) approach. This method assigns discrete copy number states, including hemizygous deletion (HETD, 1 copy), copy-neutral (NEUT, 2 copies), gain (GAIN, 3 copies), amplification (AMP, 4 copies), and high-level amplification (HLAMP, >= 5 copies). Due to resolution limitations, homozygous deletions, which tend to occur at smaller scales than 1 Mb, were not included in the analysis (*3*).

**Figure S1.** Pie chart showing the distribution of patients across treatment groups. Patients were categorized into three groups: chemotherapy + ART, chemotherapy + ART + PSMA-RLT, and chemonaïve. Of the 78 patients, 51.3% (40 patients) received chemotherapy + ART, 34.6% (27 patients) received chemotherapy + ART + PSMA-RLT, and 14.1% (11 patients) were chemonaïve.

**Figure S2.** Pie chart illustrating the distribution of radionuclide therapies administered as pre-treatment. 48.7% of patients were RLT-naïve, 33.3% received [^177^Lu]Lu-PSMA-617, 6.4% [^223^Ra]RaCl_2_, and 5.1% received combination therapies (either [^177^Lu]Lu-PSMA-617 + [^225^Ac]Ac-PSMA-617 or [^90^Y]Y-PSMA-617 + [^177^Lu]Lu-PSMA-617). The smallest group (1.3%) was treated with [^188^Re]Re-HYNIC-PSMA + [^177^Lu]Lu-PSMA-617.

**Figure S3.** ALP dynamics across [^225^Ac]Ac-/[^177^Lu]Lu-PSMA-617 treatment cycles. The x-axis represents treatment cycles from baseline (cycle 0) to treatment cycle 5, and the y-axis displays ALP levels on a logarithmic scale (log10). Box plots display the median, IQR, full range (whiskers), and individual patient values (dots).

**Figure S4.** LDH dynamics across [^225^Ac]Ac-/[^177^Lu]Lu-PSMA-617 treatment cycles. The x-axis represents treatment cycles from baseline (cycle 0) to cycle 5, and the y-axis displays LDH levels on a logarithmic scale (log10) to account for the wide variability in patient-specific LDH values. Box plots display the median, IQR, full range (whiskers), and individual patient values (dots).

**Figure S5.** Scatter plot showing the correlation between TFx (x-axis) and PSA levels (y-axis). Each red dot represents an individual patient measurement. A black linear regression line indicates the overall trend, with the shaded area representing the confidence interval to reflect data variability.

**Figure S6.** Scatter plot showing the correlation between TFx values (x-axis) and LDH levels (y-axis). Each red dot represents an individual patient measurement, capturing variability within the cohort. A black linear regression line illustrates the overall trend, with the shaded area indicating the confidence interval, reflecting uncertainty around the fit.

**Figure S7.** Scatter plot showing the correlation between TFx values (y-axis) and ALP levels (x-axis). Each red dot represents an individual patient measurement, capturing variability across the cohort. A linear regression line illustrates the overall trend, with the shaded area indicating the confidence interval and the uncertainty around the fit.

**Figure S8.** The ROC curves depict the predictive performance of pre-cycle PSA levels (black line), pre-cycle TFx (red line), and a combined model of PSA, TFx, and LDH (green line). The diagonal grey line denotes a random prediction model with an area under the curve (AUC) of 0.50, serving as a baseline for comparison. The ROC curve for pre-cycle PSA levels demonstrated no discriminative capability, with an AUC of 0.44 (95% CI: 0.29–0.59). In contrast, pre-cycle TFx exhibited improved predictive ability, achieving an AUC of 0.60 (95% CI: 0.50–0.70).

**Figure S9.** Box plot showing the distribution of TFx values across high and low CNA burden groups. The x-axis represents CNA burden categories (low = yellow vs. high = purple) while the y-axis displays TFx values on a linear scale. Boxes indicate the IQR, with the median shown as a horizontal line within each box.

**Figure S10. Gene signature comparison between baseline samples and Cluster 2.**
This heatmap illustrates the gene classification in baseline samples from 17 patients with progressive disease and in **Cluster 2**, which represents the subgroup with the poorest survival outcome. Genes are categorized according to their alteration type: **tumor suppressor deletions (blue)** and **oncogene amplifications (red)**. The plot highlights the distribution of these genomic alterations across the two groups, allowing comparison of the gene alteration patterns associated with baseline disease and the high-risk Cluster 2 profile.

**Figure S11.** EnrichR pathway analysis of oncogenes with gain-of-function alterations in Cluster 2. The x-axis represents pathway enrichment significance as -log10(p-value), highlighting the critical disruptions in DNA damage response and genomic stability.

**Figure S12:** EnrichR pathway analysis of tumor suppressor genes with loss-of-function alterations in Cluster 2. The x-axis represents pathway enrichment significance as -log10(p-value), highlighting the critical disruptions in DNA damage response and genomic stability.

**Table S1.** Association between baseline log-transformed biomarkers and TFx. Linear mixed-effects regression models were used to evaluate the association between each biomarker and tumor fraction over time.

| Biomarker (log-transformed) | 95% CI | p-value |
| --- | --- | --- |
| PSA | 0.0437–0.0793 | 2.3 × 10⁻¹⁰ |
| LDH | 0.216–0.316 | 6.3 × 10⁻¹⁹ |
| ALP | 0.085–0.169 | 4.2 × 10⁻⁸ |

*CI: confidence interval*

**References**

**1.**	Kratochwil C, Bruchertseifer F, Rathke H, et al. Targeted α-Therapy of Metastatic Castration-Resistant Prostate Cancer with (225)Ac-PSMA-617: Swimmer-Plot Analysis Suggests Efficacy Regarding Duration of Tumor Control. *J Nucl Med.* 2018;59:795-802.

**2.**	Adalsteinsson VA, Ha G, Freeman SS, et al. Scalable whole-exome sequencing of cell-free DNA reveals high concordance with metastatic tumors. *Nature Communications 2017 8:1.* 2017-11-06;8.

**3.**	Choudhury AD, Werner L, Francini E, et al. Tumor fraction in cell-free DNA as a biomarker in prostate cancer. *JCI Insight.* 2018;3.

**4.**	Fong Y, Huang Y, Lemos MP, McElrath MJ. Rank-based two-sample tests for paired data with missing values. *Biostatistics.* 2018;19:281-294.

**5.**	Obuchowski NA. Nonparametric analysis of clustered ROC curve data. *Biometrics.* 1997;53:567-578.
